# JAK inhibitors differentially modulate B cell activation, maturation and function: A comparative analysis of five JAK inhibitors in an *in-vitro* B cell differentiation model and in patients with rheumatoid arthritis

**DOI:** 10.3389/fimmu.2023.1087986

**Published:** 2023-01-26

**Authors:** Natalie Frede, Raquel Lorenzetti, Janika M Hüppe, Iga Janowska, Arianna Troilo, Marei-Theresa Schleyer, Ana C. Venhoff, Reinhard E. Voll, Jens Thiel, Nils Venhoff, Marta Rizzi

**Affiliations:** ^1^ Department of Rheumatology and Clinical Immunology, Medical Center - University of Freiburg, Faculty of Medicine, University of Freiburg, Freiburg, Germany; ^2^ Division of Rheumatology and Clinical Immunology, Medical University Graz, Graz, Austria

**Keywords:** JAK inhibition, B cells, rheumatoid arthritis, tofacitinib, baricitinib, ruxolitinib, upadacitinib, filgotinib

## Abstract

**Background:**

Janus kinase (JAK) inhibitors have been approved for the treatment of several immune-mediated diseases (IMIDs) including rheumatoid arthritis (RA) and psoriatic arthritis and are in clinical trials for numerous other IMIDs. However, detailed studies investigating the effects of different JAK inhibitors on B cells are missing. Within this study, we therefore aimed to characterize the effect of JAK inhibition on the B cell compartment.

**Methods:**

To this end, we investigated the B cell compartment under JAK inhibition and compared the specific effects of the different JAK inhibitors tofacitinib (pan-JAK), baricitinib (JAK1/2), ruxolitinib (JAK1/2), upadacitinib (JAK1/2) as well as filgotinib (selective JAK1) on *in-vitro* B cell activation, proliferation, and class switch recombination and involved pathways.

**Results:**

While B cell phenotyping of RA patients showed an increase in marginal zone (MZ) B cells under JAK inhibition, comparison with healthy donors revealed that the relative frequency of MZ B cells was still lower compared to healthy controls. In an *in-vitro* model of T-cell-independent B cell activation we observed that JAK1/2 and selective JAK1 inhibitor treatment led to a dose-dependent decrease of total B cell numbers. We detected an altered B cell differentiation with a significant increase in MZ-like B cells and an increase in plasmablast differentiation in the first days of culture, most pronounced with the pan-JAK inhibitor tofacitinib, although there was no increase in immunoglobulin secretion *in-vitro*. Notably, we further observed a profound reduction of switched memory B cell formation, especially with JAK1/2 inhibition. JAK inhibitor treatment led to a dose-dependent reduction of STAT3 expression and phosphorylation as well as STAT3 target gene expression and modulated the secretion of pro- and anti-inflammatory cytokines by B cells.

**Conclusion:**

JAK inhibition has a major effect on B cell activation and differentiation, with differential outcomes between JAK inhibitors hinting towards distinct and unique effects on B cell homeostasis.

## 1 Introduction

Rheumatoid arthritis (RA) is a chronic immune-mediated inflammatory disease, which may lead to progressive disability due to irreversible joint destruction as well as systemic complications with an elevated mortality (1, 2). B cells play an important role in the pathogenesis of RA. They accumulate in the synovium and form germinal centers, where they may differentiate into antibody-secreting plasma cells (3). Autoantibodies such as rheumatoid factor (RF) and anti-citrullinated protein antibodies (ACPA) have been shown to contribute to inflammation and bone erosions through complement and immune cell activation (4). In addition, B cells are the most important non-professional antigen-presenting cells (APC) (5). Notably, B cells are important producers of cytokines, such as pro-inflammatory TNF-alpha and IL-6, which are involved in the pathogenesis of RA and constitute already established targets of anti-cytokine directed treatments (6–9). The central role of B cells in RA is further supported by the therapeutic efficacy of rituximab, a monoclonal antibody directed against CD20 that specifically depletes B cells (10).

To date, four different Janus kinase (JAK) inhibitors (JAKi) have been successfully introduced in the treatment of immune-mediated diseases (IMIDs) such as RA, psoriatic arthritis, ankylosing spondylitis and ulcerative colitis in Europe (11–14). By interacting with different affinity with one or more of the different Janus kinases (JAK1-3 and TYK2), these small molecules affect the signalling downstream from cytokine receptors *via* Signal Transducer and Activator of Transcription (STAT) proteins. Therefore, JAK inhibition effectively reduces cytokine-mediated activation and survival of pathology-driving immune cells by targeting signalling downstream of cytokine receptors (15). Comparative studies investigating the effect of different JAK inhibitors on T cells and monocytes are available, but detailed studies on B cells are lacking. However, JAK-STAT signalling plays an important role in B cell development and function: Common-gamma-chain cytokines like IL-21, which signal through JAK1, are crucial for development from early, naïve B cells to plasmablasts (16, 17). Furthermore, IL-6 signalling involving JAK1 and JAK2 controls the survival, population expansion and maturation of B cells and plasmablasts (18, 19).

In this study, we therefore investigated the B cell compartment under JAK inhibition and compared the specific effects of the different JAK inhibitors tofacitinib (pan-JAK), baricitinib (JAK1/2), ruxolitinib (JAK1/2), upadacitinib (JAK1/2) as well as filgotinib (selective JAK1) on *in-vitro* B cell activation, proliferation, and class switch recombination and involved pathways.

## 2 Materials and methods

### 2.1 Patients and healthy donors

This study was conducted under the ethics protocol 20-1109 (ethics committee of the University of Freiburg, Germany). RA patients treated with baricitinib (Olumiant^®^, Eli Lilly), tofacitinib (Xeljanz^®^, Pfizer) or upadacitinib (Rinvoq^®^, AbbVie) were consented according to local ethics guidelines and PBMC samples were stored for B cell phenotyping. Patients’ characteristics are listed in **Table 1**. Buffy coats were purchased from the Blood Bank of the University Medical Center Freiburg (approval of the ethics committee of the Freiburg University: 147/15).

**Table 1:** Characteristics of 25 rheumatoid arthritis patients treated with JAK inhibitors.

Patient characteristics	n=25
Age, years, mean (range)	61,84 (26-82)
Sex:	
Female, n (%)	19 (76)
Male, n (%)	6 (24)
Disease duration, years, mean (range)	17.1 (1-39)
BMI, mean (range) in kg/m2	26.52 (18.99-42.28)
Nonsmokers, n (%)	22 (88)
DAS28, mean	3.27
CRP, mg/l, mean (range)	3.5 (2.98-5.35)
RF positive, n (%)	19 (76)
ACPA positive, n (%)	20 (80)
Prednisone, n (%)	15 (60)
Prednisone dose, mg/day, mean (range)	6.03 (1-15)
Methotrexate comedication, n (%)	11 (44)

BMI, Body mass index; DAS28, Disease Activity Score 28; CRP, C-reactive protein; RF, rheumatoid factor; ACPA, anti-citrullinated protein antibodies.

### 2.2 Cell isolation and culture

Peripheral blood mononuclear cells (PBMCs) were isolated from buffy coats or EDTA blood by density gradient centrifugation. Total or CD27^+^ B cells were isolated magnetically using EasySep isolation kits (Human B-cell Isolation Kit/CD27^+^ B-cell Isolation Kit (Stemcell Technologies)) following the manufacturers’ instructions. B cells were plated in 96-well plates with 30.000 cells/well in Iscove’s Modified Dulbecco’s Medium (Life Technologies) supplemented with 10% FCS, insulin, apo-transferrin, non-essential amino acids, glutamine and glutathione as described previously (20) and stimulated with CpG (ODN2009, Apara Biosciences) at a concentration of 0.5µM. Baricitinib (Toronto Research Chemicals), tofacitinib (provided by Pfizer Inc, Peaback, USA), ruxolitinib (Novartis), upadacitinib (ABT-494, MedChemExpress), filgotinib (GLPG0634, selleckchem) were stored as 10nM stocks in dimethylsulfoxide and further diluted in medium as needed. JAK inhibitors were added to indicated samples at doses of 10-3000nM.

### 2.3 Flow cytometry

Cultured B cells were stained with fluorochrome-labelled antibodies listed in **Supplemental Table 1** to assess expression of surface markers by flow cytometry (FACS Canto II, BD Biosciences). Data were analyzed with FlowJo software. To assess STAT phosphorylation, permeabilization, intracellular staining and fixation were performed following the eBioscience™ Foxp3/Transcription Factor Staining Buffer Set (ThermoFisher Scientific). In brief, cell surface markers were stained at 4°C. After washing, the cells were incubated in 100μl FoxP3 fixation/permeabilization solution for 30 min. Subsequently, intracellular antigens were stained with indicated antibodies diluted in permeabilization buffer for 30 minutes and measured by flow cytometry (FACS Canto II, BD Biosciences).

### 2.4 Phenotyping of B cell subsets *ex vivo* by flow cytometry

PBMCs were quickly thawed in a 37°C water bath, washed in warm media containing RPMI Medium with FCS and stained for 15min on ice with the antibodies listed in **Supplementary Table 1**. Dead cell exclusion was performed using a Zombie NIR Fixable Viability Kit (Biolegend). Cells were acquired using flow cytometry (Cytek Aurora, Cytek) and SpectroFlo^®^ software. The gating strategy for phenotyping the peripheral B cell compartment is shown in **Supplemental Figure 1A**.

### 2.5 Cell proliferation assay

The effect of JAK inhibition on cellular proliferation was determined by dye dilution with cell trace violet (CellTrace Violet Cell Proliferation Kit, ThermoFisher) and flow cytometric quantification of signal intensity following the manufacturer’s instructions.

Briefly, total B cells (1 × 10^6^) were suspended in 1 mL PBS and 1 μL of CellTrace Violet stock solution (5mM) was added to a final concentration of 5 μM. Cells were incubated at 37°C and protected from light for 15 min. Unbound dye was quenched by diluting with 5 volumes of complete culture medium followed by two washes with that medium. Cells were stimulated with CpG and cultured for 6 days in the presence or absence of JAKi.

### 2.6 Determination of immunoglobulin concentrations

Immunoglobulin concentrations in supernatants from *in-vitro* experiments were determined by ELISA. Nunc Maxisorb 96-well plates were coated with anti-human Ig mix (Jackson ImmunoResearch) in bicarbonate buffer and bound immunoglobulins were detected with alkaline phosphatase-conjugated anti-human IgG/IgM/IgA (Jackson ImmunoResearch) using p-nitrophenyl phosphate (Sigma-Aldrich) in DEA buffer as a substrate. Ig concentrations were calculated by the interpolation of calibration curves with Ig standard (N Protein Standard SL; Siemens).

### 2.7 Cytokine multiplex assay

Cytokine secretion was measured in supernatants of *in-vitro* experiments with a bead-based immunoassay using the LEGENDPlex (BioLegend) pre-defined human B cell panel following the manufacturer’s instructions. Samples were read by flow cytometry (FACS Canto II, BD Biosciences).

### 2.8 Quantitative PCR

RNA was extracted using Qiagen’s RNEasy kit and transcribed into cDNA with SuperScript III reverse transcriptase (Invitrogen) and random hexamer primers (Amersham Pharmacia Biotech). Quantitative PCR was performed on a StepOnePlus Real Time PCR machine (Applied Biosystems) using TaqMan Gene Expression Master Mix (Applied Biosystems) and ThermoFisher TaqMan Assay probes.

### 2.9 Statistical analysis

Statistical analysis was performed on absolute measurements rather than CpG normalized values with the help of GraphPad Prism 8 (GraphPad Software, La Jolla, CA), using ANOVA with Dunnett’s post-test or Student’s t-test.

## 3 Results

### 3.1 Patient characteristics

25 RA patients treated with the JAK inhibitors baricitinib (16 patients), tofacitinib (8 patients) or upadacitinib (1 patient) were included. The mean age at assessment was 61.8 years (range 26-82 years) with a mean disease duration of 17.1 years (range 1-39 years). Within this cohort, 19 patients were positive for RF and 20 for ACPA. 11 patients received a JAK inhibitor in combination with methotrexate (MTX), while 14 patients were under JAKi monotherapy. Mean CRP serum concentration at timepoint of assessment was 3.5mg/l (range 2.98-5.35mg/l). Patients had on average moderate disease activity with a mean disease activity score 28 (DAS28) of 3.27. A detailed overview of patients’ characteristics can be found in **Table 1**. Patients were compared to 30 healthy donors, mean age of healthy donors was 53.5 years and 60% were male and 40% female.

### 3.2 B cell phenotyping of RA patients under JAK inhibition

**Figure 1:**
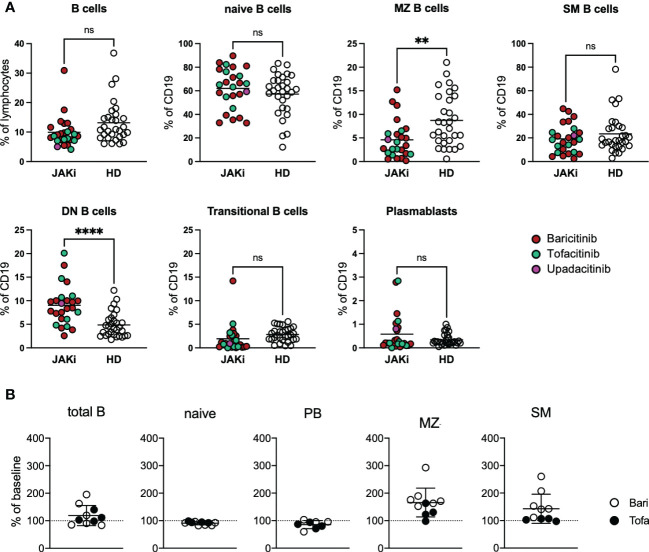
B cell phenotyping of RA patients under JAK inhibitor treatment. **(A)** PBMCs from 25 RA patients under JAK inhibitor treatment (16 baricitinib, 8 tofacitinib, 1 upadacitinib) as well as 30 healthy donors were stained with fluorochrome-labelled antibodies to assess expression of surface markers by spectral flow cytometry (Cytek Aurora). Gating strategy depicted in **Supplementary Figure 1A**. Statistical analysis by t-test, **p < 0.005, 0.0005, ****p < 0.0001; ns, not significant. **(B)** B-cell subpopulations of 10 RA patients before and under JAK inhibitor therapy. Data depicted as percent of baseline. PB, plasmablasts; MZ-like, marginal zone-like B cells; SM, switched memory B cells; Bari, baricitinib; Tofa, tofacitinib.

RA patients under JAK inhibition showed a statistically non-significant decrease in B cell frequency compared to healthy donors. Averages of overall B cell counts were within normal range (mean 159 cells/µl, range 46.4-431 cells/µl). Regarding B cell subpopulations, RA patients under JAK inhibition showed significantly lower MZ B cells than healthy donors, whereas double negative B cells (DN, IgD-CD27-) were significantly increased in the patient group (**Figure 1A**). BAFF receptor (BAFFR) surface expression was reduced compared to healthy donors, whereas TACI surface expression did not differ significantly. Regarding activation markers, CD95 expression was significantly lower in patients than in healthy controls (**Supplementary Figure 1B**). IgG1-4, IgM and IgA1/2 surface expression on B cells did not differ significantly between patients under JAK inhibition and healthy donors (**Supplementary Figure 1C**). Furthermore, there were no significant differences regarding B cell subpopulations between patients receiving baricitinib or tofacitinib (data not shown). Also concomitant anti-rheumatic therapy with methotrexate or prednisolone statistically did not make a difference regarding B-cell subpopulations (data not shown).

For 10 patients treated with JAK inhibitors we were able to compare B cell subpopulations before start of JAK inhibitor treatment and under treatment (**Figure 1B**). JAKi treatment resulted in a slight increase of mean total B cell numbers (from mean 167 cells/µl to 185 cells/µl). We furthermore observed a relative expansion of marginal-zone (MZ) B cells with JAKi treatment in nine out of ten patients (mean 166%, range 98-293%, p=0.0032, **Figure 1B**) under JAK inhibitor treatment, which, however, did not lead to normalization of MZ B cell numbers to levels comparable to healthy donors (see **Figure 1A**, p=0.0031).

### 3.3 *In-vitro* model of T-cell independent B cell activation

**Figure 2:**
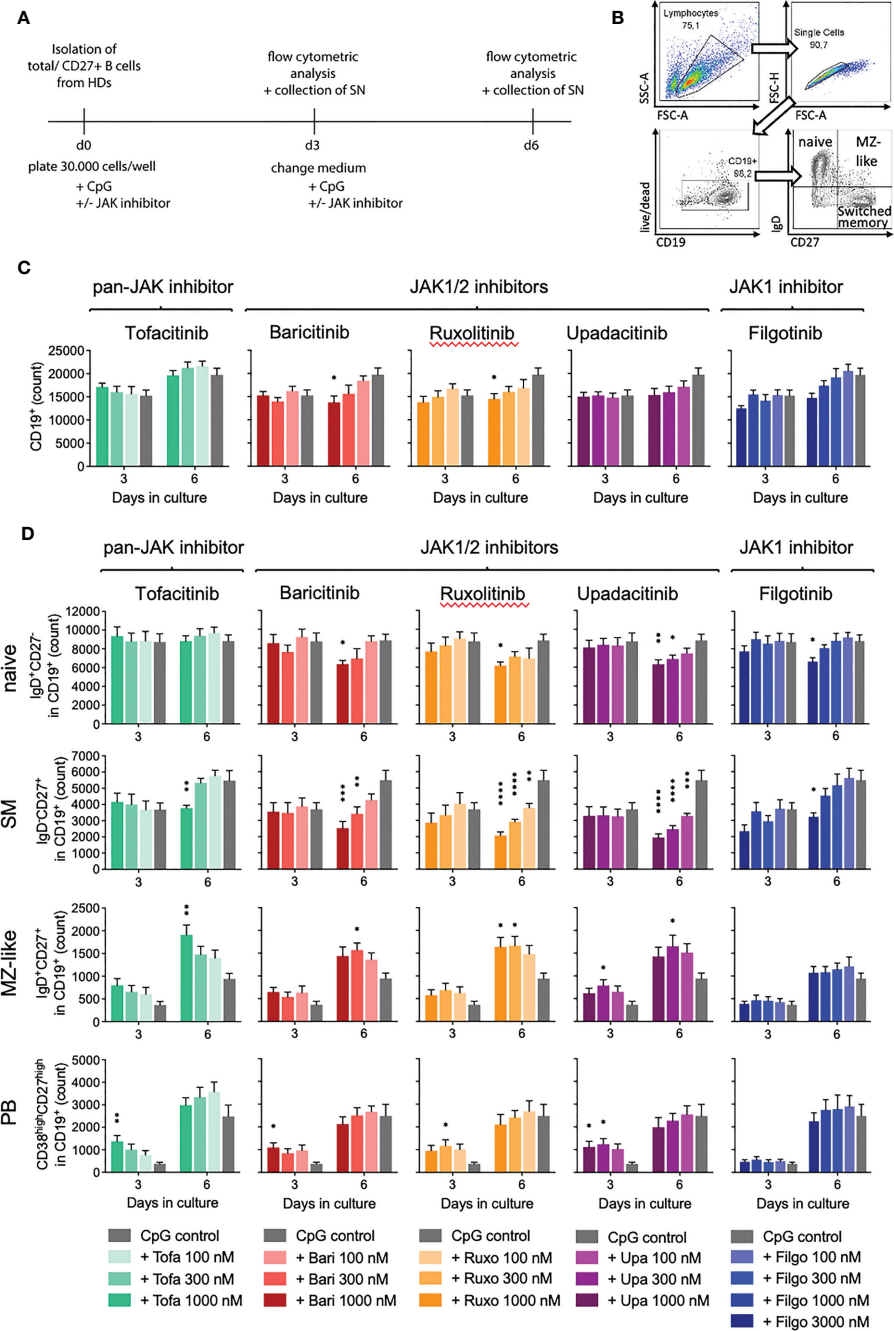
Impact of JAK inhibition on B cell differentiation in T-independent *in-vitro* culture. Primary total B cells were activated with CpG on day 0 and cultured in the absence or presence of indicated concentrations of JAK inhibitors. On day 3 and 6, B cell subpopulations were analyzed by time acquisitioned flow cytometry. **(A)**: Experimental set-up for *in-vitro* culture model of T-cell-independent B cell activation with CpG, known to support MZ B cell expansion. **(B)**: Gating strategy. Lymphocytes were defined by the cell size/granularity. After exclusion of doublets and dead cells, live CD19+ cells were defined as B cells. Among these, naive, MZ, and memory B cells were defined by IgD and CD27 expression and plasmablasts by expression of CD38 and CD27. **(C)**: Total B cell count depicted in absence or presence of JAK inhibitors. Data shown as mean ± SEM of 4 independent experiments, with triplicates each; **(D)**: Absolute numbers of naive (IgD+CD27-), memory (IgD-CD27+), and MZ B cells (IgD+CD27+), as well as plasmablasts (CD38highCD27high) within the live/CD19+ population. Data shown as mean ± SEM of 4 independent experiments, each in triplicate. ANOVA with Dunnett’s multiple comparisons test as follow-up test. *p < 0.05, **p < 0.005, ***p < 0.0005, ****p < 0.0001 indicate significant differences of JAK inhibitors compared to CpG control.

To investigate the observed expansion of MZ B cells in RA patients under JAKi treatment, we turned to an *in-vitro* model of T-cell-independent B cell activation with CpG *via* toll-like receptor 9 (TLR9), known to support MZ B cell expansion (**Supplementary Figure 2** illustrates B cell activation through CpG). JAK1/2 (baricitinib, ruxolitinib, upadacitinib) as well as selective JAK1 inhibitor treatment (filgotinib) led to a time- and dose-dependent decrease of total B cell numbers compared to the CpG control (baricitinib > ruxolitinib > upadacitinib > filgotinib), whereas the pan-JAK inhibitor tofacitinib did not influence total B cell counts significantly (**Figures 2A–C**).

### 3.4 JAK inhibition results in altered B cell differentiation

When assessing B cell subpopulations, we observed an altered B cell differentiation with JAKi treatment *in-vitro* (**Figure 2D**). Specifically, naïve B cells, which represent the majority of total B cells, were significantly reduced on day 6 of culture in samples treated with 1000nM, respectively 3000nM (filgotinib) doses of JAK1/2 and selective JAK1 inhibitors (**Figure 2D**, upper panel, p=0.0411 for baricitinib and p=0.0044 for upadacitinib, respectively). Pan-JAK inhibitor tofacitinib did not significantly alter naïve B cell numbers.

**Figure 3:**
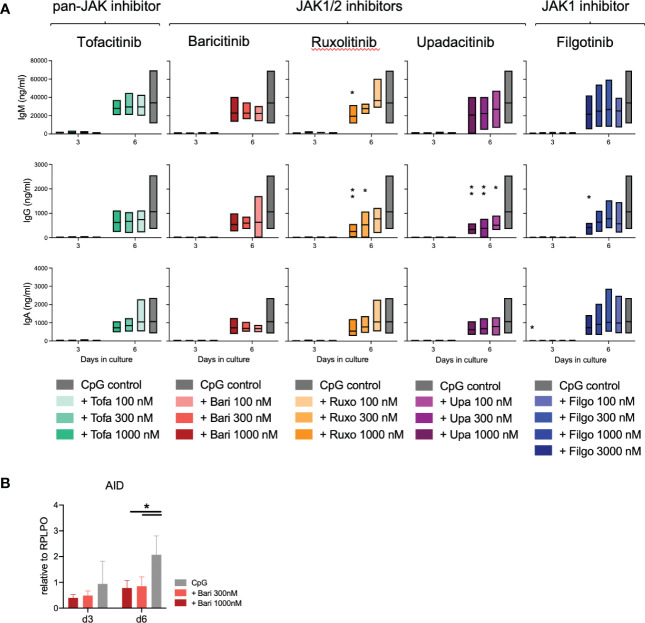
Immunoglobulin secretion and class switch under JAK inhibition. **(A)**: Immunoglobulins measured by ELISA in supernatants of 3 experiments with triplicates each. Data depicted as floating bars (min to max) with line at mean. ANOVA with Dunnett’s multiple comparisons test as follow-up test, *p < 0.05, **p < 0.005. **(B)**: Expression of the class-switch mediating enzyme AID under JAK inhibition with baricitinib measured by qPCR. Data from 4 experiments depicted relative to B cell housekeeping gene RPLPO. ANOVA with Dunnett’s multiple comparisons test, *p < 0.05.

Notably, we further detected a highly significant dose- and time-dependent reduction of switched memory (SM) B cells, strongest with JAK1/2 inhibition (upadacitinib > ruxolitinib > baricitinib > tofacitinib > filgotinib). There was a greater than 50% reduction of SM B cells in samples treated with 1000nM concentrations of upadacitinib, ruxolitinib and baricitinib, while the selective JAK1 inhibitor filgotinib only led to a significant decrease at a dose of 3000nM (**Figure 2D**). Consistent with these findings, we observed a decreased expression of Activation-induced Cytidine Deaminase (AID) in treated samples, an essential enzyme for somatic hypermutation and class-switch (**Figure 3B**).

Interestingly, we additionally observed a significant increase in MZ-like B cells under JAK inhibition compared to the CpG control (**Figure 2D**, mean 161%, range 118-212%). This effect was more pronounced upon pan-JAK inhibitor treatment than JAK1 or JAK1/2 inhibition (tofacitinib > upadacitinib > ruxolitinib > baricitinib). Selective JAK1 inhibition with filgotinib did not have any significant effect on MZ-like B cell numbers.

Concomitantly, we detected an increase in plasmablast differentiation in the first three days of culture (**Figure 2D**, mean 258%, range 138-369%). This effect was again strongest under pan-JAK inhibitor tofacitinib (tofacitinib > upadacitinib > ruxolitinib > baricitinib). We did not observe an expansion of plasmablasts under filgotinib treatment, consistent with our observations in MZ-like B cells. On day 6 of culture there were no significant differences in plasmablasts between JAK inhibitor-treated samples and the CpG control (**Figure 2D**). Exemplary plots are shown in **Supplementary Figure 3**.

When measuring immunoglobulins in the supernatant, there was a slightly increased IgM production on day 3 of culture (tofacitinib), however this was not statistically significant (**Figure 3A**). On day 6 of culture, immunoglobulins in the supernatant were dose-dependently reduced under JAK inhibition, especially IgG secretion was significantly reduced in upadacitinib- and ruxolitinib-treated samples (**Figure 3A**, p=0.0014, respectively 0.0013).

### 3.5 Expansion of MZ-like B cells due to proliferation

**Figure 4:**
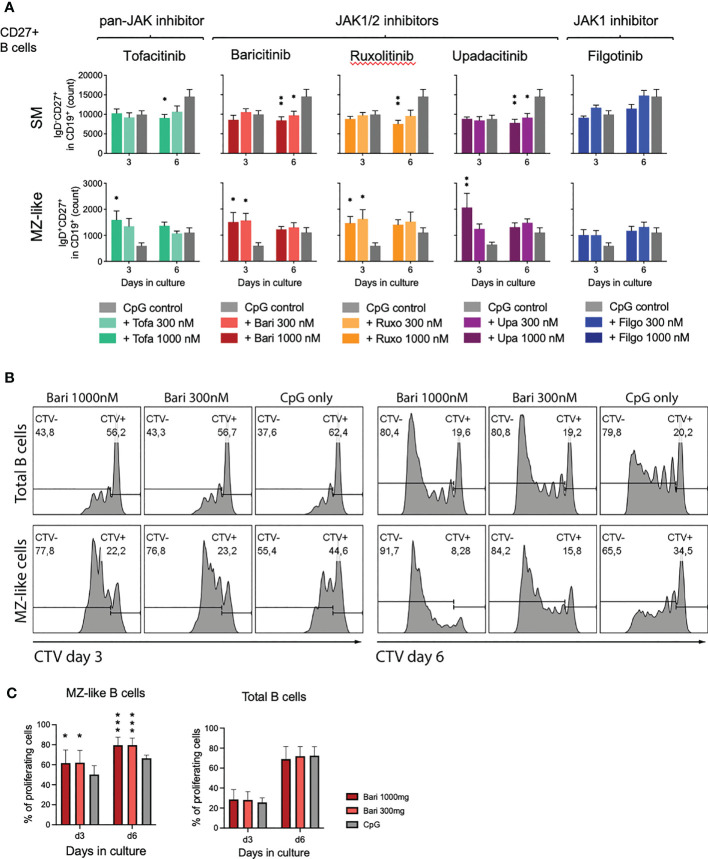
Expansion of MZ-like cells due to proliferation of pre-existing cells. **(A)**: Isolated CD27+ B cells were activated with CpG on day 0 and cultured in the absence or presence of indicated concentration of JAK inhibitors. On day 3 and 6, B cell subpopulations were analyzed by time acquisitioned flow cytometry. Data shown as mean ± SEM of 3 independent experiments, each in triplicate. ANOVA with Dunnett’s multiple comparisons test as follow-up test. *p < 0.05, **p < 0.005 indicate significant differences of JAK inhibitors compared to CpG control. For gating strategy and exemplary plots also see **Supplementary Figure 4**. (B+C): Cell Trace Violet (CTV) stained, primary total B cells were activated with CpG on day 0 and cultured in the absence or presence of indicated concentration of baricitinib. B cells were analyzed by flow cytometry on days 3 and 6. Exemplary plots depicted, CTV-: proliferating B cells; CTV+: non-proliferating B cells **(B)**. Three independent experiments, performed in triplicates, student’s t-test. *p < 0.05, ***p < 0.0005 indicate significant differences of JAK inhibitors compared to CpG control **(C)**.

To assess whether the observed effect of MZ-like B cell expansion was due to generation of MZ-like cells from naïve B cells or proliferation of existing MZ-like B cells, we isolated CD27+ B cells (including MZ and SM B cells) and cultured these with CpG and scalar doses of JAK inhibitors. We observed significant MZ-like B cell expansion with all JAK inhibitors apart from filgotinib (**Figure 4A**, lower panel, mean 278%, range 177-338%), suggesting proliferation of pre-existing MZ-like cells. These results were validated by assessing proliferation of both, total B cells as well as individual subpopulations under JAK inhibition through Cell Trace Violet staining. Indeed, MZ-like B cells had a higher proliferation rate under JAK inhibitor treatment with baricitinib compared to the CpG only control (**Figures 4B, C**). In baricitinib-treated samples 79.60%, respectively 79.54% of MZ-B cells had divided at least once after 6 days in culture vs 66.52% in CpG control (p=0.0005, **Figure 4C**).

### 3.6 JAK inhibition results in reduced STAT3 expression and basal phosphorylation

**Figure 5:**
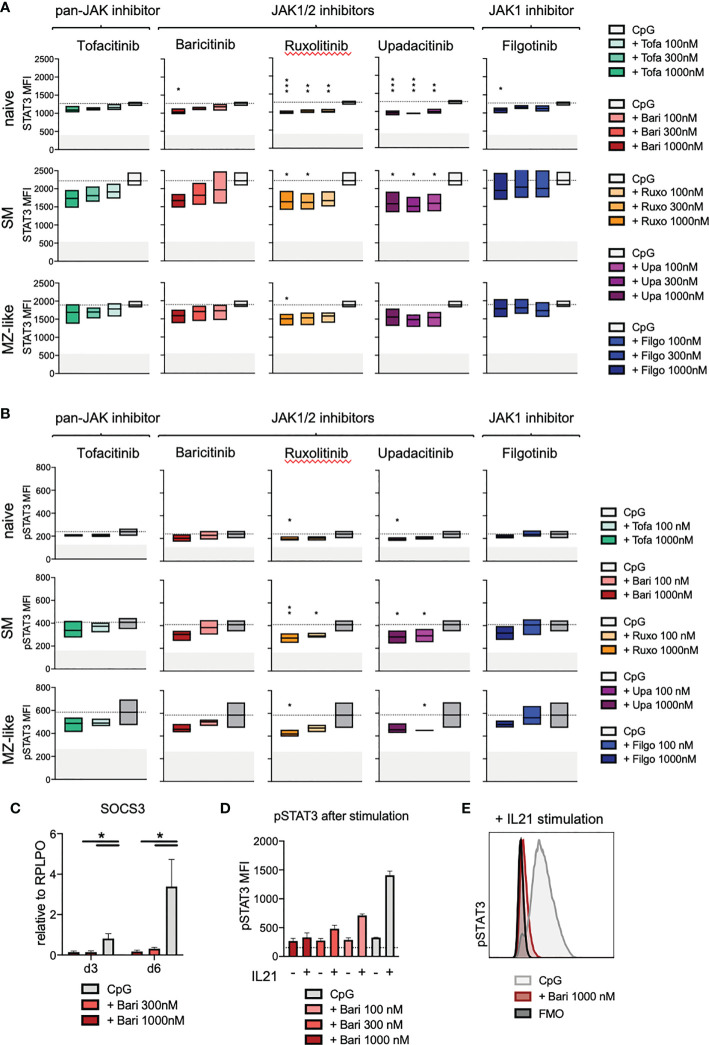
STAT3 expression and phosphorylation under JAK inhibitor treatment. Primary total B cells stimulated with CpG on day 0 and treated with scalar doses of JAK inhibitors as indicated, intracellular staining performed on day 3 of culture. **(A)**: STAT3 expression in CD19+ B cells analyzed by flow cytometry. Data of 3 independent experiments, depicted as floating bars (min to max) of STAT3 mean fluorescence intensity. ANOVA with Dunnett’s multiple comparisons test as follow-up test. *p < 0.05, **p < 0.005, ***p < 0.0005, indicate significant differences of JAK inhibitors compared to CpG control. **(B)**: Basal phosphorylation (without further stimulation) of STAT3 in CD19+ B cells, analyzed by flow cytometry. Data of 3 independent experiments, with duplicates depicted as floating bars (min to max) with line at mean. ANOVA with Dunnett’s multiple comparisons test as follow-up test, *p < 0.05, **p < 0.005 indicate significant differences of JAK inhibitors compared to CpG control. **(C)**: Expression of STAT3-target gene SOCS3 under baricitinib treatment assessed by qPCR. Data from 4 experiments depicted relative to B cell housekeeping gene RPLPO. ANOVA with Dunnett’s multiple comparisons test, *p < 0.05. **(D, E)**: STAT3 phosphorylation under baricitinib treatment upon additional stimulation of B cells with IL-21. IL-21 was added to indicated wells 15 minutes before fixation of cells, otherwise as detailed above.

In order to investigate the downstream effects of JAK inhibition on the JAK/STAT signalling pathway in total B cells as well as individual B cell subpopulations, we assessed STAT3 and 5 expression and phosphorylation, known to be important in B cells. JAK inhibitor treatment led to a dose-dependent reduction of STAT3 expression especially in naïve, but also in switched memory (SM) and MZ-like B cells on day 3 of culture (ruxolitinib > upadacitinib > baricitinib > filgotinib > tofacitinib) (**Figure 5A**, for gating strategy and exemplary plots see **Supplementary Figure 5**). In line with these findings, we observed a reduced basal STAT3 phosphorylation (without additional stimulation) in all B cell subpopulations upon JAK inhibition, suggesting that signalling downstream of JAKs was significantly reduced (**Figure 5B**). Expression of STAT3-target gene SOCS3 was greatly diminished under baricitinib treatment with doses of 300nM and 1000nM (**Figure 5C**, p=0.0132, respectively p=0.0271 for day 3 and day 6), suggesting that downstream signalling was immediately and almost completely abrogated. This was consistent with a strong dose-dependent reduction of STAT3 phosphorylation upon further stimulation of B cells with IL-21 under baricitinib treatment (**Figures 5D, E**). Results for both, STAT5 expression and phosphorylation, were not significant, though there was a tendential slight increase in both STAT5 expression and basal phosphorylation under JAK inhibition (**Supplementary Figures 6A, B**). In case of additional stimulation with IL-21, JAK inhibition with baricitinib led to a dose-dependent reduction of STAT5 phosphorylation in naïve B cells but not in total B cells (**Supplementary Figure 6C**).

### 3.7 JAK inhibition leads to changes in cytokine secretion

**Figure 6:**
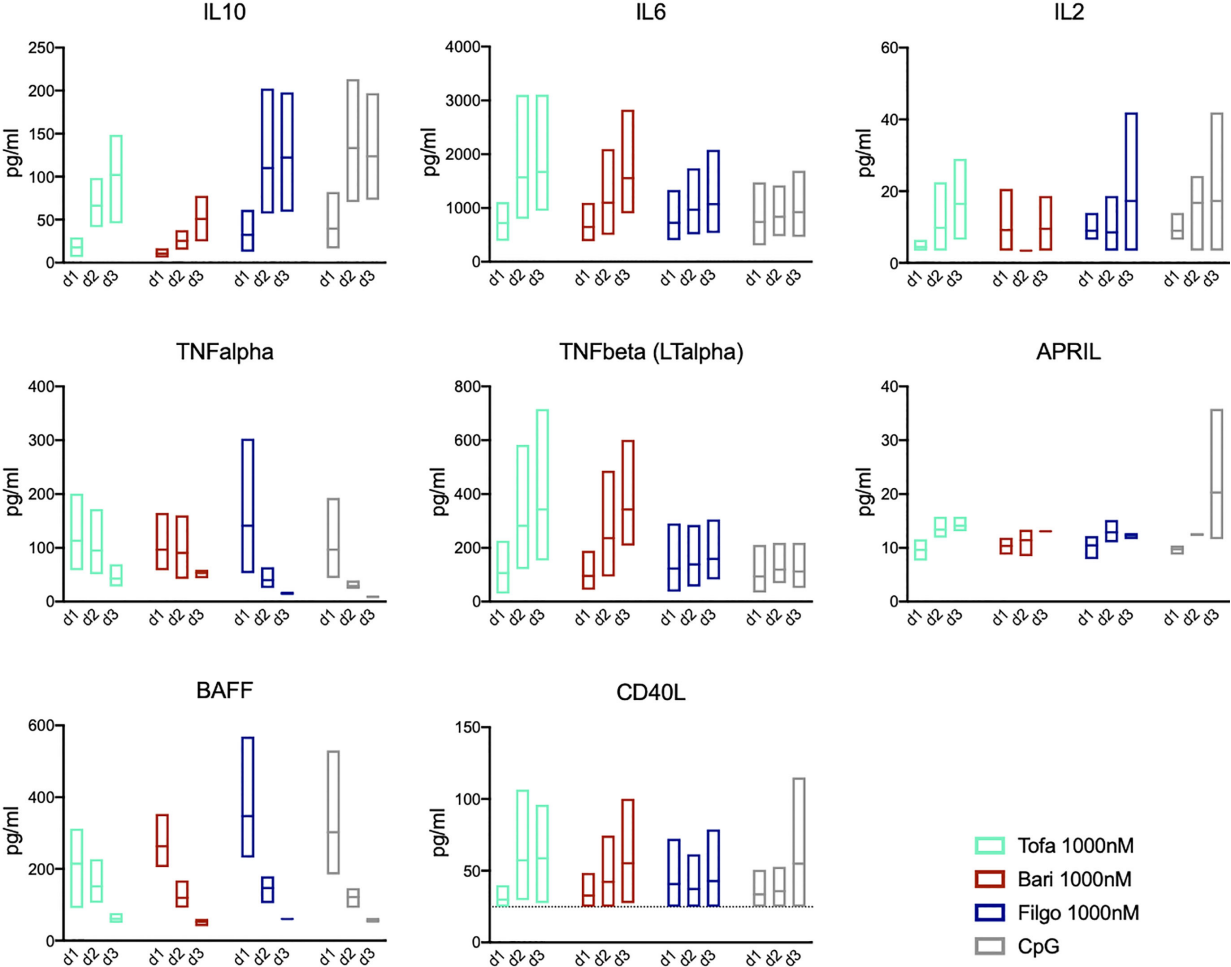
JAK inhibition leads to altered cytokine secretion profile. Secretion of IL-10, IL-6, IL-2, TNF-α, TNF-ß (LT-α), APRIL, BAFF, and CD40L was measured in supernatants of *in-vitro* B cell culture by cytokine multiplex assay on day 1, 2, and 3 during treatment with Tofacitinib, Baricitinib or Filgotinib. Data depicted as floating bars (min to max) with line at mean.

To assess the role of autocrine signalling, we measured cytokine secretion of the cultured B cells on days 1-3 after CpG stimulation. In this T-cell-independent culture system B cells produced IL-10, IL-6, TNF-α, lymphotoxin-α (LT-α), APRIL, BAFF as well as small amounts of CD40L and IL-2 after CpG stimulation. IL-10 secretion increased to peak on day 2/3, whereas TNF-α and IL-6 decreased or remained stable, respectively. BAFF secretion decreased rapidly over days 1-3, whereas APRIL secretion increased with time (**Figure 6**). Upon treatment with JAK1/2 inhibitor baricitinib as well as pan-JAK inhibitor tofacitinib, IL-10 secretion was reduced on days 1-3 compared to the CpG control (p=0.0115 on day 2). This in turn resulted in increased IL-6 and LT-α production as well as a delayed downregulation of TNF-α, demonstrating that the cytokine profile is modulated by JAK inhibition. Selective JAK1 inhibition through filgotinib had little effect on IL-10, IL-6, TNF-α and LT-α production. All JAK inhibitors led to a tendentially decreased production of APRIL on day 3 compared to the CpG control, although not statistically significant. Downregulation of BAFF secretion was slightly delayed in pan-JAK inhibition with tofacitinib. These findings indicate that JAK inhibition broadly affects the kinetics/dynamics and amounts of cytokine secretion of both, JAK-dependent but also JAK-independent cytokines.

## 4 Discussion

JAK-STAT signalling plays an important role in B cell development and function. In this study, we compared the specific effect of the pan-JAK inhibitor tofacitinib, the JAK1/2 inhibitors baricitinib, ruxolitinib, and upadacitinib and the selective JAK1 inhibitor filgotinib on *in-vitro* B cell activation, development and proliferation. In a T-cell-independent *in-vitro* B cell differentiation model, JAK inhibition led to a reduced total B cell number as well as reduced development of switched memory B cells, whereas MZ-like B cells were increased. Especially JAK1/2 inhibition strongly impaired switched memory formation in a time- and dose-dependent manner. In addition, JAK inhibition also led to changes in cytokine secretion dynamics and amounts, potentially impacting other cell types.

In collagen-induced arthritis (CIA), a widely accepted mouse model of rheumatoid arthritis, MZ B cells have been described to play an important role in the initiation of arthritis (21). However, MZ B cells also seem to play a role in the downregulation of disease and induction of remission in the CIA model through secretion of anti-inflammatory IL-10 (22, 23). In general, in mice MZ B cells are thought to have immunoregulatory functions and play a role in prevention of autoimmunity (24). The role of MZ(-like) B cells in human RA is less clear. However, also human MZ B cells are known to be important producers of immunomodulatory IL-10 (24) and MZ B cells have been reported to be reduced in autoimmune vasculitis and SLE (24, 25). In this study, we also observed decreased MZ B cells in RA patients, with tendential improvement under JAK inhibitor treatment. However, the investigated patient cohort was heterogenous regarding age, used JAK inhibitor as well as concomitant anti-rheumatic therapy (e.g. methotrexate, prednisone). Further studies will be necessary to elucidate the role of MZ B cells in RA as well as in autoimmune disease in general.

CpG, which was used for stimulation in the cell culture model in this study, is known to induce the NF-κB and MAPK pathways, leading to secretion of cytokines by the B cells, such as IL-2, IL-6, IL-10, and TNF-α (26, 27). These secreted cytokines act on the B cells in an autocrine manner, driving activation, proliferation and differentiation (28). The different B cell subpopulations rely on different cytokine signals for activation, proliferation and differentiation (29), which may explain the observed alterations in B cell differentiation. MZ B cells for example express high levels of TACI and can be activated by BAFF and APRIL, i.e. TNF superfamily cytokines not reliant on JAK/STAT signalling (30). Furthermore, MZ and SM B cells are known to express higher levels of TLR9, and thus can be more readily activated by CpG stimulation than naïve B cells (31).

Indeed, the observed early increase of plasmablasts under JAK inhibition likely results from MZ and memory B cells, which express high levels of TLR9 and are known to rapidly generate plasmablasts without the need for T-cell help (32). We did not observe significant differences in antibody concentrations in supernatants on day 3, which can be attributed to the fact that antibody production is still low on day 3. However, we observed a tendentially reduced antibody production on day 6 of culture under JAK inhibition.

Many cytokines can induce antibody secretion with, however, different efficacy and specificity (33). Especially IL-21 and IL-10 are potent inducers of antibody production (34). It thus can be hypothesized that blocking cytokine signalling, particularly IL-10 signalling, which relies on JAK1/TYK2 and STAT3, by JAK inhibitor treatment leads to reduced immunoglobulin production. The crucial role of STAT3 in cytokine-mediated antibody secretion is also underlined by the fact that naïve B cells from AD-HIES patients with mutations in STAT3 cannot differentiate into antibody-secreting cells upon IL-21-/IL-10 stimulation and patients display absence of antibody-secreting long-lived plasma cells (35).

Furthermore, we observed a strong decrease of SM B cells in JAK1/2 inhibition. This may be explained by the fact that JAK1/2 inhibitors effectively block IL-6, IL-10 and IL-21 signalling and lead to reduced STAT3 expression and phosphorylation, which plays an important role in generation and expansion of memory B cells (29, 36). This hypothesis is supported by the fact that loss of function mutations of STAT3, the IL-21 receptor (IL21R) as well as the IL-6 receptor chain IL6R all lead to impaired memory B cell formation with reduced memory B cell numbers (29, 35, 37, 38). The autocrine effects of JAK-independent cytokines may thus drive the observed differential activation and proliferation of B cell subpopulations and subsequently contribute to further changes in the cytokine secretion profile.

We investigated primarily STAT3 expression and phosphorylation as STAT3 is known to play an important role on B cell activation and maturation. Furthermore, TLR stimulation through CpG is known to lead to JAK-independent STAT3 activation (39, 40). Recently, also STAT5 was implicated in the maintenance of B cell homeostasis (41), thus we also investigated the effect of JAK inhibition on STAT5 expression and phosphorylation. However, while STAT5 plays an important role in IL-21-mediated (T-cell dependent) B cell activation, especially of naïve B cells (41), its role in T-cell independent B cell activation seems limited.

In recent years it became evident that cross-talk between the JAK/STAT pathway and other signalling pathways plays an important role in activating and regulating immune cells. For instance, NF-κB, PI3K/Akt and MAPK pathways constitute important signalling pathways in B cell activation and differentiation (42–47). Thus, effects of JAK inhibition may not be restricted to the classical JAK/STAT pathway, but JAK inhibition might also exert beneficial effects on other inflammatory signalling cascades. STAT3 in particular plays an important role in regulating other B cell transcription factors, especially transcription factors driving antibody-secreting cell differentiation, such as BLIMP1 and IRF4 (48, 49). Further studies will be needed to investigate the impact of JAK inhibitors on non-canonical JAK/STAT signalling.

Our results show differential outcomes for the different JAK inhibitor classes. While originally licensed as a selective JAK1 inhibitor, upadacitinib had effects similar to the JAK1/2 inhibitors baricitinib and ruxolitinib rather than to the selective JAK1 inhibitor filgotinib. These findings are in line with those of McInnes et al., who reported a strong activity also against JAK2 for upadacitinib in *in-vitro* experiments (50), which stands in contrast to initial *in-vitro* kinase assays indicating both upadacitinib and filgotinib to be highly selective JAK1 inhibitors (51–54). Also Traves et al. recently reported the highest JAK1 selectivity for filgotinib when comparing tofacitinib, baricitinib, upadacitinib and filgotinib, indicating that upadacitinib, baricitinib and tofacitinib each had significant additional effects on mainly JAK2 or JAK3 dependent pathways. Potencies depended on cytokine stimulation, STATs used for readout and assessed cell type (55). Overall, in our study upadacitinib, respectively tofacitinib, had the most potent effects on B cell activation and terminal differentiation, which is in line with McInnes’ and Traves’ results, who also reported the strongest *in-vitro* activity for upadacitinib and tofacitinib (50, 55). Data on direct clinical comparisons of the different JAK inhibitors are missing. However, a network meta-analysis of three JAK inhibitors licensed for RA (baricitinib, tofacitinib and upadacitinib) by Pope et al. showed that upadacitinib had a higher efficacy regarding ACR response and achievement of remission than the other JAK inhibitors (56), and other network meta-analyses gave similar results (57–59).

In general, more selective JAK inhibitors are considered to have less side effects. Especially first- generation JAK inhibitors may lead to hematological side effects thought to derive from JAK2/3 inhibition, laboratory alterations of liver enzymes and cholesterol/lipid levels as well as an increased risk of thrombosis (53, 60). However, the most commonly reported side effects of JAK inhibitors are infections, including respiratory and urinary tract infections as well as opportunistic and viral infections with an increased risk of varicella zoster virus (VZV) reactivation (60–62). In controlled clinical trials filgotinib did not cause a marked increase in herpes zoster, which was in contrast to other JAKi (63). Whether side effects will differ significantly between the different JAK inhibitors in real-world application, however, remains to be seen.

Whether there are any significant differences between the different JAK inhibitors specifically regarding B cell function in clinic also remains to be seen. The follow-up time of the recently approved substances is still too short to compare long-term effects and JAK inhibitors are just now being investigated for predominantly B-cell mediated autoimmune diseases, such as systemic lupus erythematodes.

JAK inhibition may, however, lead to a reduced vaccination response. Previously, data had been published for tofacitinib, baricitinib and upadacitinib, demonstrating a slightly reduced response to pneumococcal but not influenza vaccines for tofacitinib as well as a mild impairment of tetanus but not pneumococcal vaccine response for baricitinib (64–66). In times of the COVID19 pandemic this topic has gained new interest. Recently, Seror et al. published data from the French MAJIK-registry, showing that upadacitinib treatment led to lower antibody titers and a higher rate of vaccine non-responders upon COVID19 vaccination compared to baricitinib and tofacitinib (67). Furer et al. described a slight but significantly reduced immunogenicity of BNT162b2 mRNA COVID-19 vaccine in adult patients with rheumatic diseases and JAKi treatment (68). It is known that for the establishment of long-lasting antibody responses IL-21 signalling through STAT3 is essential, which was severely depressed by JAK inhibitors in our study (35), giving a mechanistic explanation for reduced antibody response.

Regarding overall immunoglobulin levels under JAK inhibition, only few published data are available. For baricitinib, tofacitinib and upadacitinib decreases in mean or median immunoglobulin levels were reported from clinical trials, though values were still within normal ranges (69–71). Similarly, for filgotinib, serum IgG, IgM and IgA levels remained within normal range in phase 3 clinical trials (72). Long-term follow-up data on immunoglobulin levels under JAK inhibition are missing. Among patients investigated in this study, two out of 25 patients developed hypogammaglobulinemia under JAK inhibitor treatment, which may, however, also be related to concomitant therapy as well as previous therapies. In our cell culture model, especially IgG secretion was significantly reduced upon upadacitinib and ruxolitinib treatment. Further studies will be needed to elucidate the full impact of JAK inhibition on the physiological B cell response and antibody production with special focus on humoral response to neoantigens and the development of immunoglobulin diversity and antibody repertoire.

In conclusion, we demonstrated that JAK inhibition has major effects on B cell activation and maturation, with differential outcomes between individual JAK inhibitors hinting towards distinct and unique effects on B cell homeostasis. These data contribute to a better understanding of the mechanism of action of JAK inhibitors in treatment of antibody-dependent autoimmune conditions. Increased knowledge of the individual effects and side effects of JAK inhibitors will facilitate an individualised medicine approach as well as the development of novel therapeutic agents with less side effects.

## Data availability statement

The raw data supporting the conclusions of this article will be made available by the authors, without undue reservation.

## Ethics statement

The studies involving human participants were reviewed and approved by Ethics committee of University of Freiburg, Freiburg, Germany. The patients/participants provided their written informed consent to participate in this study.

## Author contributions

NV, MR, RV, and JT participated in the design and supervision of the study and gave critical input. NF, RL, JH, IJ, TA, and AV performed experiments. RV, NV, JT, NF, and M-TS carried out patient recruitment and consenting, patient sample collection and provided clinical information. NF, RL, and JH performed data analysis and interpretation. NF, RL, MR, JT, RV, and NV wrote the manuscript. All authors contributed to the article and approved the submitted version.
