# Supplementary material for: JAK inhibitors differentially modulate B cell activation, maturation and function: A comparative analysis of five JAK inhibitors in an in-vitro B cell differentiation model and in patients with rheumatoid arthritis
Source: Front Immunol. 2023 Jan 26;14:1087986. doi: 10.3389/fimmu.2023.1087986 (PMC9908612; doi:10.3389/fimmu.2023.1087986)
Supplement: Supplementary file 1 [file DataSheet_1.docx]

Supplementary Material

**Supplementary Table 1: Antibodies**

| **Marker** | **Labelling** | **Supplier** |
| --- | --- | --- |
| CD3 | Super Bright 436 | Invitrogen |
| CD16 | Super Bright 436 | Invitrogen |
| CD19 | APC-Cy7 | BioLegend |
| CD20 | Pacific Blue | BioLegend |
| CD21 | Alexa Fluor 700 | BioLegend |
| CD27 | Brilliant Violet 421 | BioLegend |
| CD27 | BV786 | BD Bioscience |
| CD27 | PerCP/Cy5.5 | BioLegend |
| CD33 | Super Bright 436 | Invitrogen |
| CD38 | PE-Cy7 | BioLegend |
| CD38 | PerCP | BioLegend |
| CD38 | BV650 | BioLegend |
| CD69 | BV480 | BD Bioscience |
| CD86 | BV711 | BioLegend |
| CD95 | BV421 | BD Bioscience |
| CD138 | PE-Cy7 | BioLegend |
| CD268 (BAFF-R) | BV605 | BD Bioscience |
| IgA | APC | Jackson Immuno |
| IgA | PE | Southern Biotech |
| IgA1 | PerCP-Cy5.5 | Cytognos |
| IgA2 | PerCP-Cy5.5 | Cytognos |
| IgA1 | APC | Cytognos |
| IgD | FITC | Southern Biotech |
| IgD | PerCP-eFluor 710 | Invitrogen |
| IgD | PE-Cy7 | BioLegend |
| IgG | APC | Jackson Immuno |
| IgG1 | PE | Cytognos |
| IgG2 | PE | Cytognos |
| IgG2 | FITC | Cytognos |
| IgG3 | FITC | Cytognos |
| IgG4 | APC | Cytognos |
| IgM | FITC | BioLegend |
| IgM | Alexa Fluor 647 | Jackson ImmunoResearch |
| live/dead | BV510 (AmCyan) | BioLegend |
| live/dead | APC-H7 | BioLegend |
| STAT1 | PE | BD PhosFlow |
| pSTAT1 (pY791) | Pacific Blue | BD |
| STAT3 | APC | BD PhosFlow |
| pSTAT3 (pY705) | PE | BD Biosciences |
| STAT5 | FITC | Invitrogen |
| pSTAT5 (pY694) | PE-Cy7 | BD PhosFlow |
| TACI | PE-Dazzle594 | BioLegend |

## Supplementary Figures:


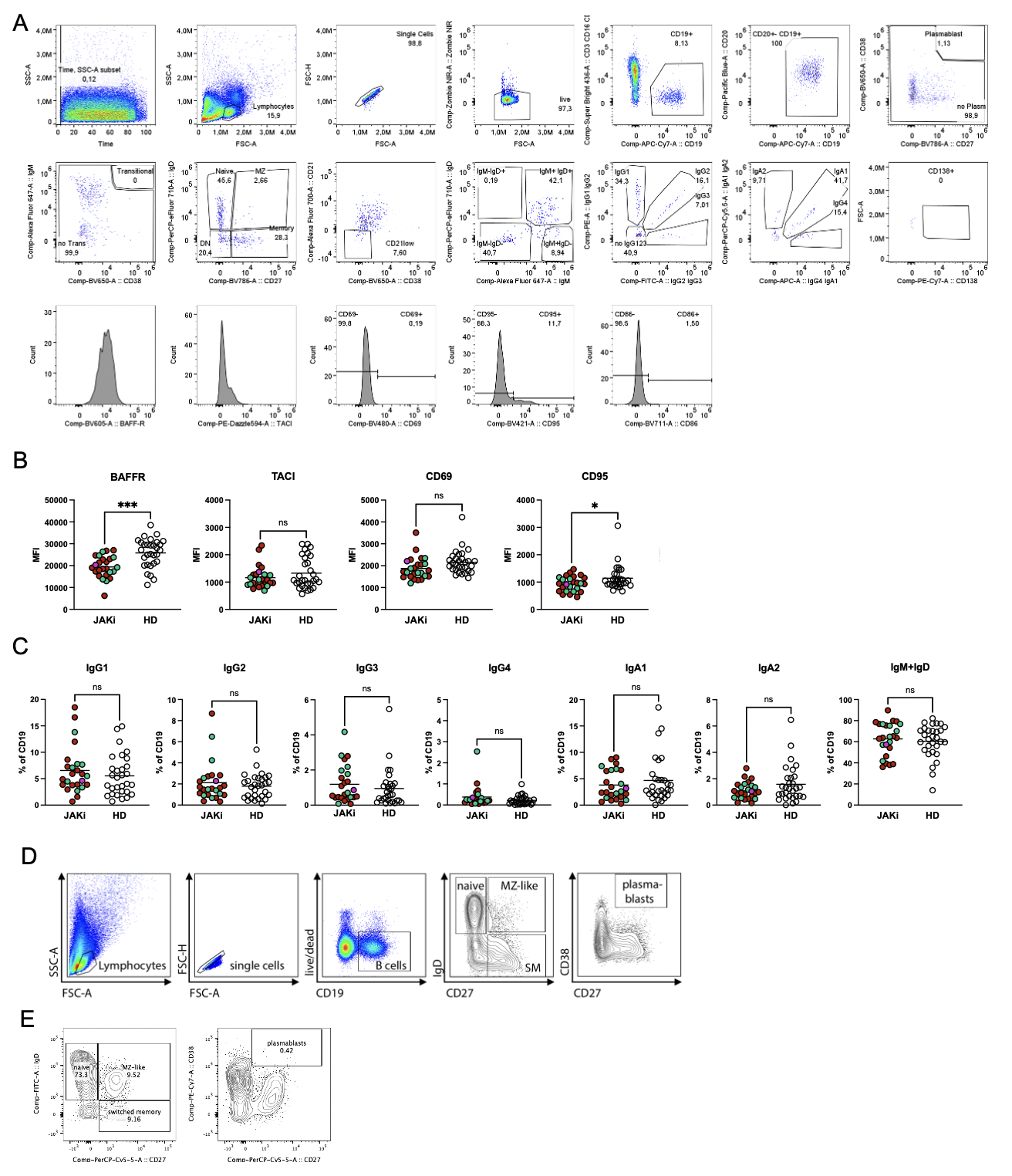


**Supplemental Figure 1: B cell phenotyping of RA patients under JAK inhibition.** Related to Figure 1. (A): Gating strategy for characterisation of patient B cells under JAK inhibitor treatment. (B): B-cell surface expression of activation markers and B-cell receptors BAFF-R and TACI in RA patients under JAK inhibitor treatment (25) compared to healthy donors (30). Statistical analysis by t-test, *p<0.05, ***p<0.0005 compared to healthy donors (HD), ns not significant. (C): Immunoglobulin surface expression on B cells of RA patients (25) treated with JAK inhibitors, ns not significant. (D): Gating strategy for Figure 1B. (E): Exemplary plots showing B cell subpopulations of a patient. Naïve, MZ-like and switched memory B cells were identified within the CD19-positive population by expression of IgD and CD27, whereas plasmablasts were identified by expression of CD27 and CD38.

**
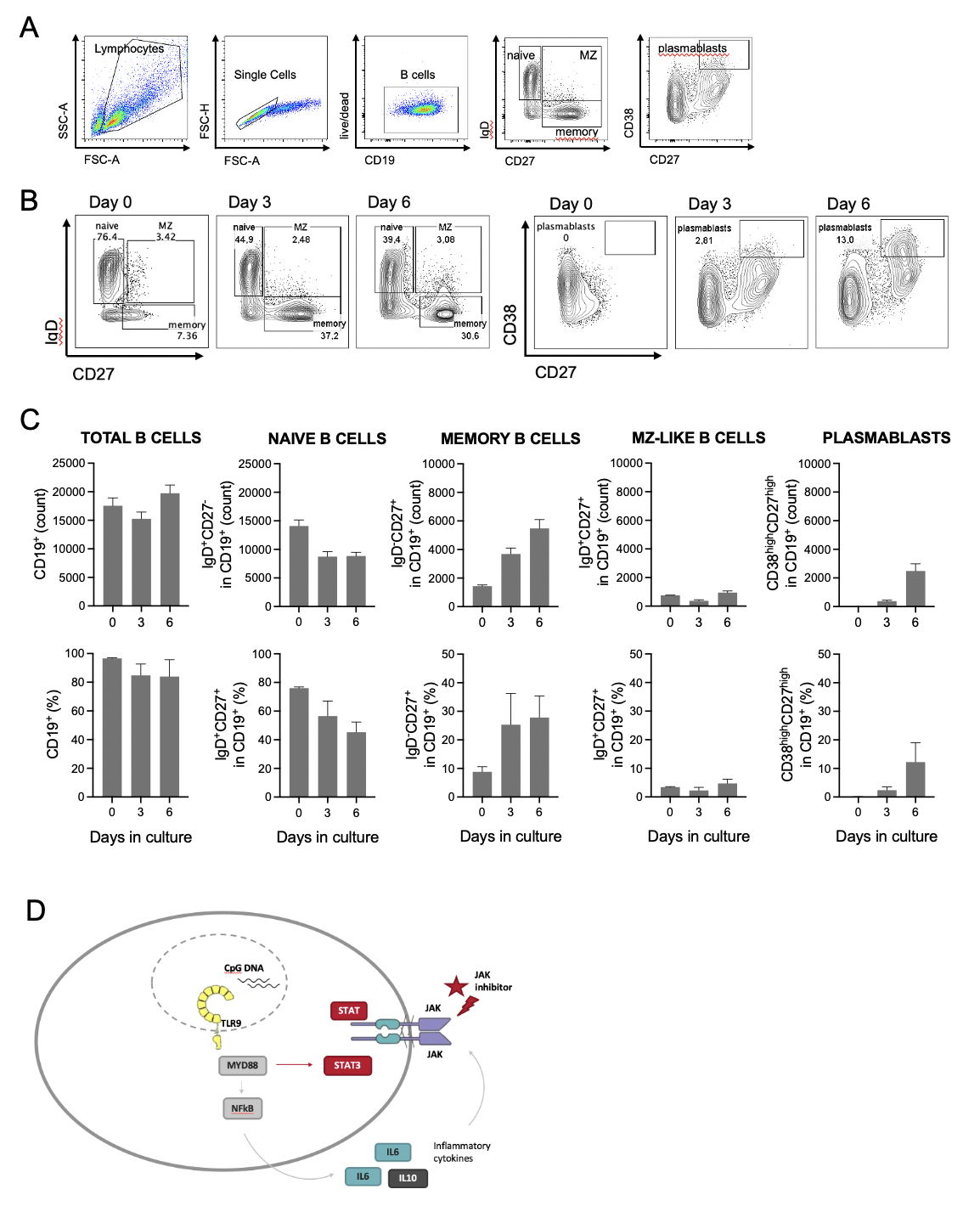
**

**Supplemental Figure 2: Effect of CpG stimulation on B cells.** Primary total B cells were activated with CpG on day 0. On day 3 and 6, B cell subpopulations were analyzed by time acquisitioned flow cytometry. **(**A): Gating strategy. (B) Exemplary plots showing naïve, MZ and memory B cell subpopulations on days 0, 3 and 6 identified by expression of IgD and CD27 as well as plasmablasts identified by CD27/CD38 expression. (C): Total B cells and B cell subpopulations shown in counts (upper panel) and percentage (lower panel) on days 0, 3 and 6 of culture. (D): Schematic drawing showing CpG stimulation of B cell. CpG is sensed by TLR9, which signals through MyD88 and activates the NfKB pathway. The latter leads to production of various cytokines, eg. IL6 and IL10, which may in turn stimulate the B cells in an autocrine manner through activation of the JAK-STAT pathway. Furthermore, CpG stimulation leads to JAK-independent STAT3 activation.

**
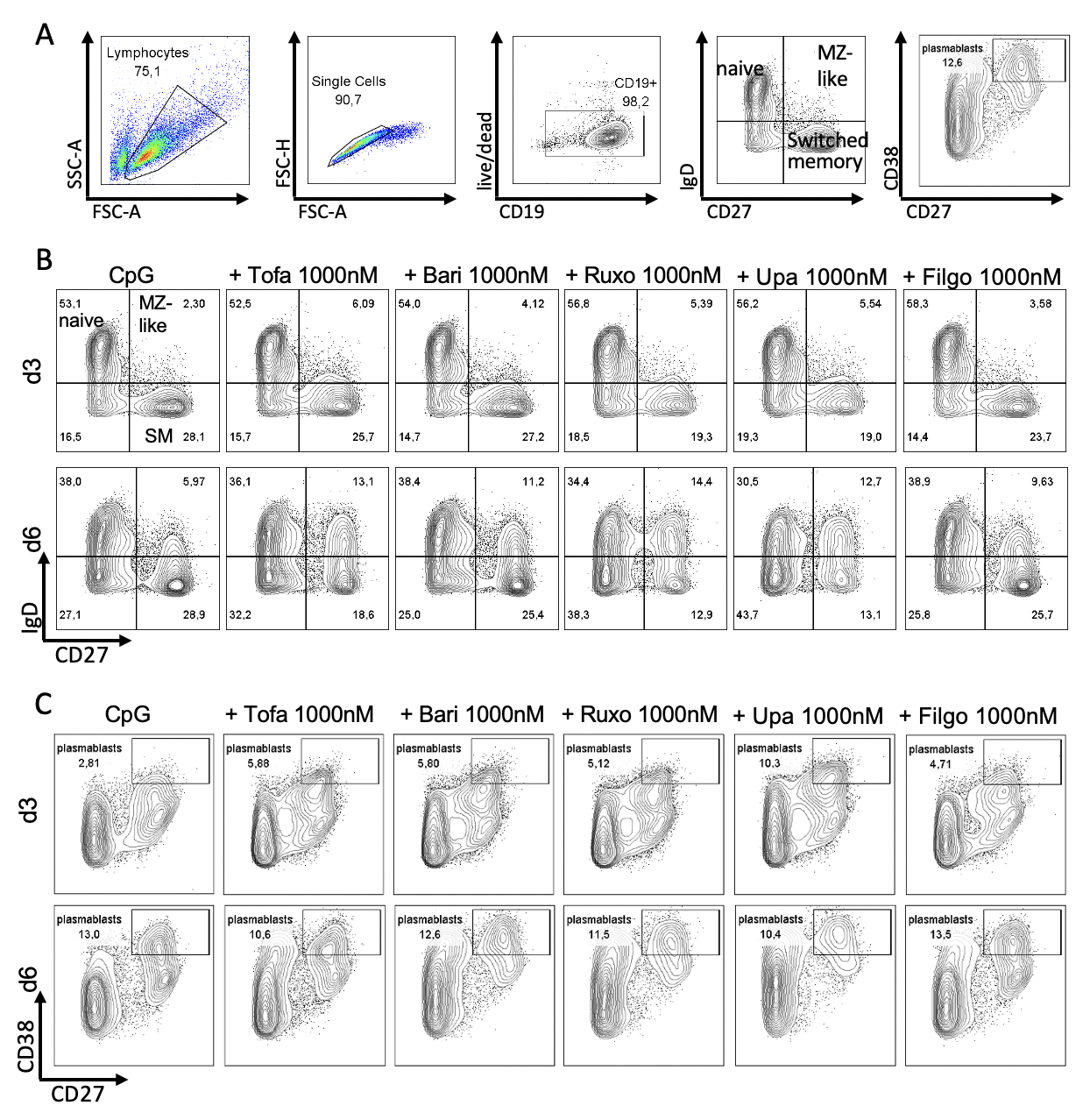
**

**Supplemental Figure 3: JAK inhibition leads to altered B cell differentiation.** Related to Figure 2. Primary total B cells were activated with CpG on day 0 and cultured in the absence or presence of different JAK inhibitors. On day 3 and 6, B cell subpopulations were analyzed by flow cytometry. (A): Gating strategy. (B): Exemplary plots showing B cell subpopulations. Within the live CD19+ population, naive B cells were characterized as IgD+CD27-, MZ B cells as IgD+CD27+, and memory B cells as IgD-CD27+. (C): Plasmablasts were characterized as CD27^high^CD38^high^ within the live CD19+ population.


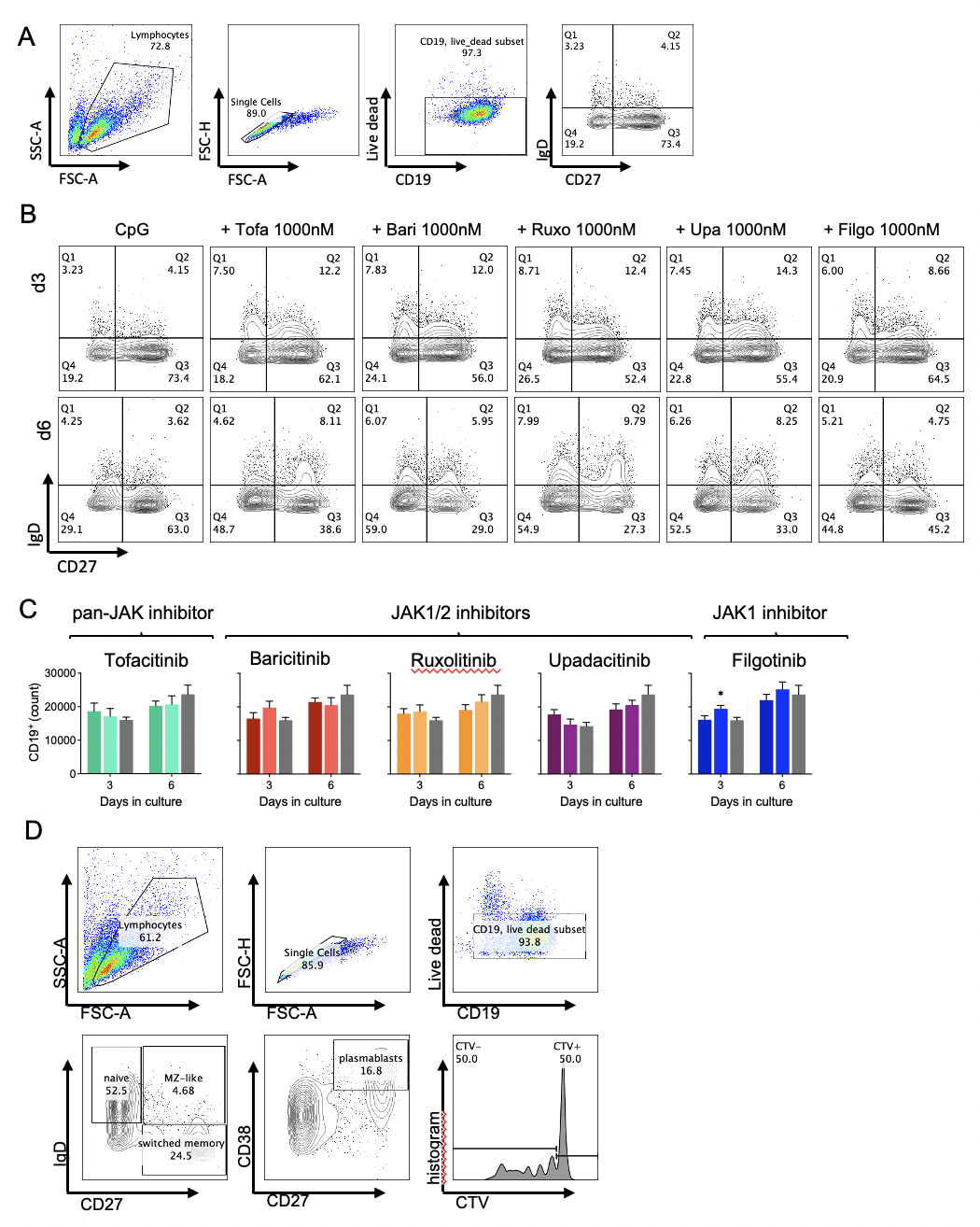


**Supplemental Figure 4: Expansion of MZ-like cells due to proliferation of pre-existing cells.** Related to Figure 4. (A): Gating strategy for CD27+ B cell experiments, shown in Figure 4A. Isolated CD27+ B cells were activated with CpG on day 0 and cultured in the absence or presence of indicated concentration of JAK inhibitors. On day 3 and 6, B cell subpopulations were analyzed by time acquisitioned flow cytometry. (B): Exemplary plots showing B cell subpopulations. Within the live CD19+ population, naive B cells were characterized as IgD+CD27-, MZ B cells as IgD+CD27+, and memory B cells as IgD-CD27+. (C): Total B cell count depicted in absence or presence of JAK inhibitors. Data shown as mean ± SEM of 3 independent experiments, with triplicates each; ANOVA with Dunnett‘s multiple comparisons test as follow-up test. *p<0.05 indicates significant differences of JAK inhibitors compared to CpG control. D: Gating strategy for CTV experiments (see Figures 4B+C).


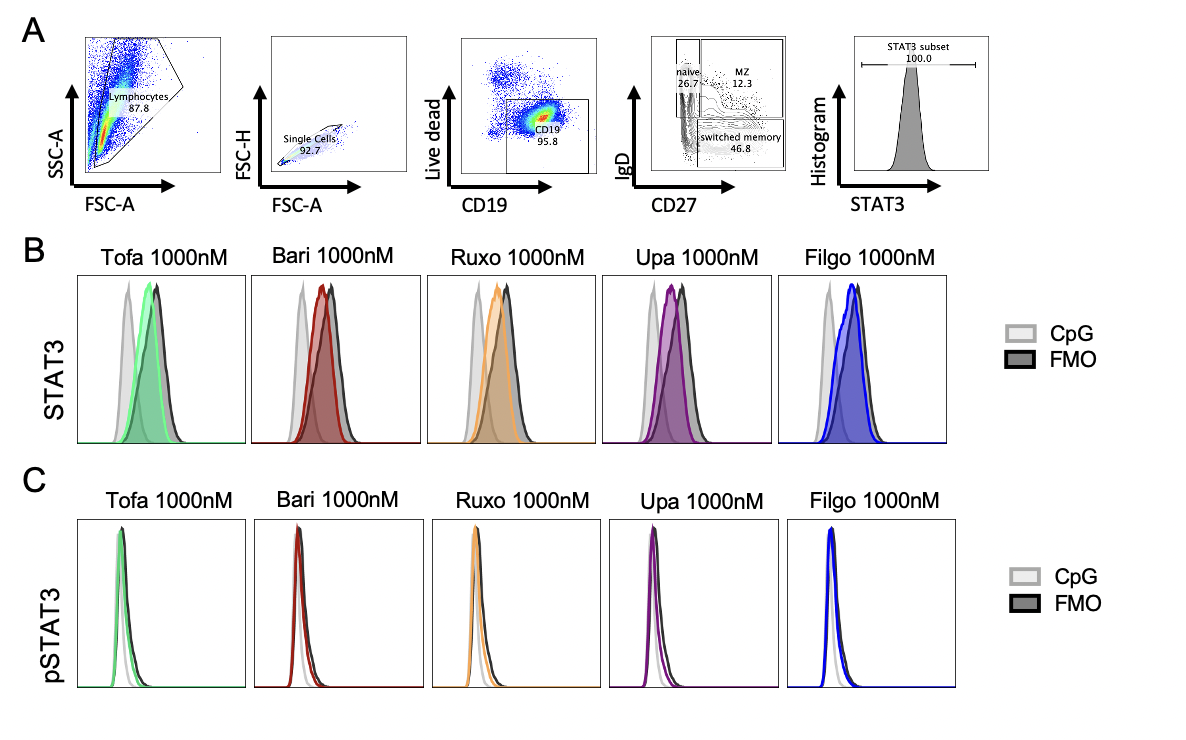


**Supplemental Figure 5: Gating strategy and STAT3 expression and phosphorylation.** Related to Figure 5. **(**A): Gating strategy for STAT expression and phosphorylation experiments, shown in Figure 5. (B+C): Shown in grey is CpG stimulated control, in dark grey FMO (fluorescence minus one) and in colour cells treated with indicated JAK inhibitors. Exemplary plots showing STAT3 expression of total B cells (CD19+ population) as histograms. (C): Exemplary plots showing STAT3 phosphorylation of total B cells (CD19+ population) as histograms.


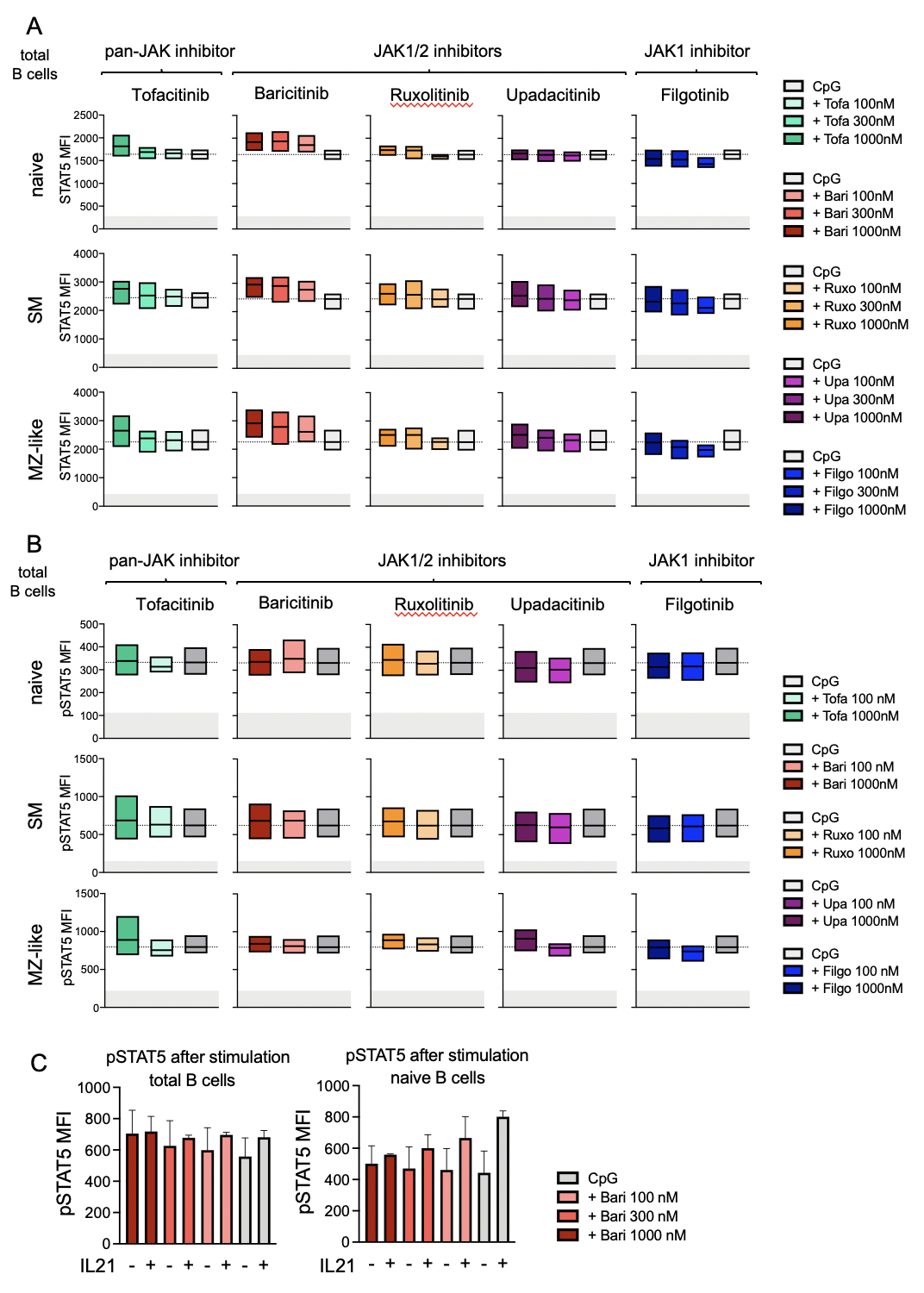


**Supplemental Figure 6: STAT5 expression and phosphorylation under JAK inhibitor treatment.** Primary total B cells stimulated with CpG on day 0 and treated with scalar doses of JAK inhibitors as indicated, intracellular staining performed on day 3 of culture. (A): STAT5 expression in CD19+ B cells analyzed by flow cytometry. Data of 3 independent experiments, depicted as floating bars (min to max) of STAT5 mean fluorescence intensity. ANOVA with Dunnett‘s multiple comparisons test as follow-up test. (B): Basal phosphorylation of STAT5 without additional stimulation in CD19+ B cells was analyzed by flow cytometry. Data of 3 independent experiments, with duplicates depicted as floating bars (min to max) with line at mean. ANOVA with Dunnett‘s multiple comparisons test as follow-up test. (C): STAT5 phosphorylation under baricitinib treatment upon additional stimulation of B cells with IL-21. IL-21 was added to indicated wells 15 minutes before fixation of cells, otherwise as detailed above.
